# Surface-Coated Nano-Sized Aluminum Powder’s Applications in Explosives and Propellants: A Review

**DOI:** 10.3390/nano15171295

**Published:** 2025-08-22

**Authors:** Weipeng Zhang, Huili Guo, Weiqiang Pang

**Affiliations:** Xi’an Modern Chemistry Research Institute, Xi’an 710065, China

**Keywords:** nano-sized aluminum powder, surface coating, modification, explosives and propellants

## Abstract

Aluminum powder has the advantages of high calorific value, high density and convenient source, and is a commonly used metal fuel in the explosives and propellants industry. Nanometer aluminum powder (nAl) has higher reactivity and higher reaction completeness than micron aluminum powder (μAl), which can improve the energy performance of mixed explosives and the burning rate of propellant. However, nAl has some disadvantages, such as easy oxidation and deterioration of the preparation process, which seriously affect its application efficiency. In order to improve these shortcomings, suitable surface coating treatment is needed. The effects of surface coating on the characteristics of nAl and on the energy and safety of explosives are summarized in this paper. The results show that surface coating of nAl can not only improve the compatibility between nAl and energetic materials, reduce the hygroscopicity of energetic composites, mitigate the easy oxidation of nAl, and protect the preparation process, but also improve the energy performance of explosives and the burning rate of propellant, increase the reaction characteristics of energetic mixtures, and reduce the mechanical sensitivity of those mixtures. In addition, the surface coating modification of nAl can obviously reduce the agglomeration of condensed-phase combustion products, thus reducing the loss of propulsion efficiency caused by agglomeration. This study is expected to provide reference for the surface coating of nAl and its application in explosives.

## 1. Introduction

Aluminum powder is the most important metal fuel in the explosives industry, as it can greatly improve the energy performance of explosives. At present, the most commonly used aluminum powder is micron-grade. Nano-aluminum powder (nAl) has a larger specific surface area because of its smaller particle size, and can be used as a high-energy fuel in explosives to further improve their energy performance. Many studies have found that due to the higher reactivity and completeness of nAl, the application of an appropriate amount of nAl can improve the work power of mixed explosives, the overpressure peak of the air explosion shock wave, or the underwater explosion energy [1,2,3]. It can significantly improve the burning rate and the characteristics of combustion products [4,5] when used in propellant.

However, due to its large specific surface area, nAl is easily oxidized, and its application efficiency will be seriously affected if it is stored or applied improperly. When aluminum powder is oxidized or burned, it will form a surface oxide layer, which will hinder the internal aluminum powder from continuing to react. Furthermore, the application of nAl in explosives will increase the viscosity of the system and worsen the preparation process of mixed explosives or solid propellants [6]. Suitable surface coating modification treatment is needed in order to address these shortcomings.

The latest research progress of nAl modification technology is summarized in this paper with regard to the influence of surface coating modification on the performance of nAl, the energy of explosives and propellants, the safety performance of explosives, and the technological performance and other properties of explosives, with the aim of providing technical support for the explosives and propellants industry.

It should be pointed out that the research ideas contained herein are relatively immature and the research content imperfect, as this is a new research direction and has therefore presented few research results. Even so, some directional views can still be drawn, which can guide future research directions and content.

## 2. Effect of Surface Coating on nAl Properties

Surface coating of nAl can protect the activity of nAl and increase its potential reaction heat, facilitating reaction and increasing the reaction degree, making the nAl reaction more complete.

### 2.1. Protecting the Activity of nAl

**Figure 1:**
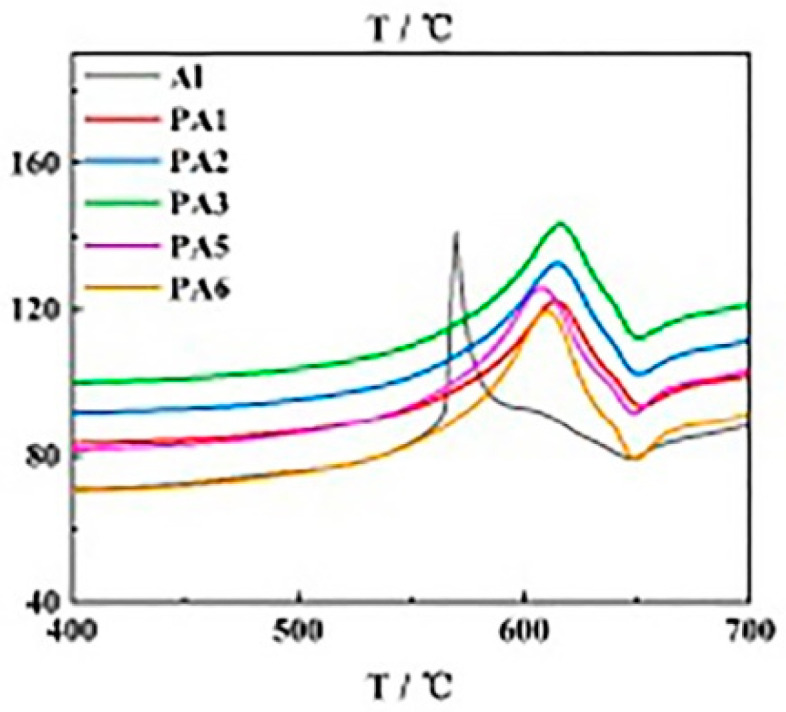
DSC results of nAl coated by PEG with different contents (the PEG contents of PA1–PA6 are 2%, 1%, 0.6%, 0.3%, 0.2%, and 0.05% respectively).

Surface coating modification can effectively protect the activity of nAl and increase its application potential. There are many methods to characterize the activity (active aluminum content) of nAl. For example, titration can directly measure the active aluminum content of nAl and thus directly judge the change of active aluminum of nAl. The delay of DSC or DTA exothermic peak indicates that nAl will react more completely at higher temperature. For the protection of nAl activity by surface coating, Zhang [7] prepared nAl@PS microcapsules by surface coating with polystyrene and analyzed the activity of aluminum powder in microcapsules stored for different lengths of time. It was found that the active aluminum content was 76.07%, 76.06%, and 74.81%, respectively, after 30 days in sealed storage and oxygen tank under natural conditions, indicating that nAl@PS can maintain its activity over a long period. In contrast, nAl without surface coating showed only 42.3% of the active aluminum content when stored naturally under the same conditions. In addition, Hao and others used esters such as hydroxyl-terminated polybutadiene-toluene diisocyanate (HTPB-TDI), epoxy resin, trimethylolpropane triacrylate ((CH_2_=CHCOOCH_2_)_3_CCH_2_CH_3_) (TMPTA) [8,9,10,11,12,13], long-chain hydrocarbons such as paraffin wax [11], organic acids such as perfluorotetradecanoic acid, palmitic acid [11,12], and amines like octadecylamine (ODA) [10] to surface-coat nAl, all of which significantly improved the long-term storage performance of highly active nAl. In particular, Kwon [14] used boron to coat nAl (nAl@B), and stored it in air with 70% humidity for one year; the active aluminum content of nAl@B particles decreased from 84% to 82%, indicating that boron provided excellent surface coating effects. Because the boron coating can increase the combustion heat of nAl at the same time, this coating strategy has significant reference value. Luo [15] used polyethylene glycol (PEG) with different contents to coat nAl (nAl@PEG), and the results of differential scanning calorimetry (DSC) are shown in Figure 1. It can be seen that the peak temperature of thermal decomposition of nAl@PEG samples has shifted backward to some extent, indicating that PEG has an obvious protective effect on the activity of nAl.

The above studies show that the oxidation rate of nAl can be greatly reduced by using appropriate surface coating materials and processes, especially by using boron powder to coat nAl, which can improve the energy effect of aluminum powder and may have a high application potential.

### 2.2. Improving the Reactivity of nAl

**Figure 2:**
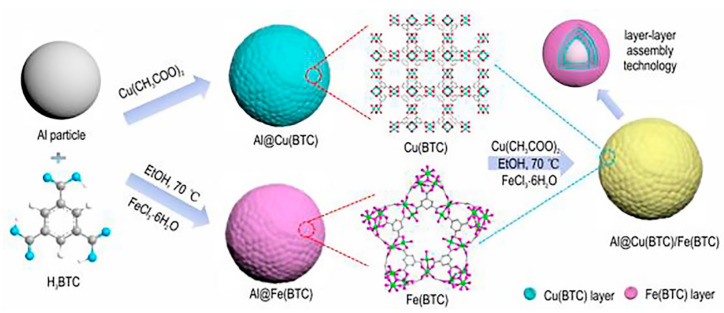
Diagram of the preparation route to core–shell-structured nAl@Cu(BTC)/Fe(BTC) nano-thermite [21].

Reactivity has a significant impact on the application efficiency of explosives and propellants. Generally speaking, the stronger the reactivity, the easier it is for nAl to react, which facilitates the complete release of energy from nAl and enhances its application efficiency. The reactivity of nAl can be characterized by the initial reaction temperature, ignition success, ignition temperature, ignition delay time, instantaneous heat flux rate, flame intensity, and peak flame temperature. Zhang [16] prepared nAl-coated carbon surfaces using laser-induced composite heating, resulting in a core–shell-structured material with particle sizes ranging from 20 to 60 nm and shell thicknesses of 3 to 8 nm. Differential thermal analysis (DTA) revealed that this core–shell-structured material began to release heat at 400 °C, far below the melting point of aluminum (660 °C), and more than 100 °C earlier than nAl coated on alumina surfaces. Yao [17] prepared nAl by using perfluoro tetradecanoic acid (PTA) to coat nAl (nAl@PTA) with an average particle size of 50 nm and purity greater than 98.0% using a surface coating agent content of 10 wt%. Laser ignition tests were conducted and compared with uncoated aluminum powder. The study found that compared to uncoated aluminum powder, the ignition delay time was shorter, the combustion reaction was more intense, and the flame brightness was higher when the laser thermal flux density was lower. Combustion tests also showed that the combustion of coated aluminum powder was more complete. Wang [18] prepared ammonium perchlorate (AP) surface-coated nAl using the recrystallization method, with the surface coating agent content ranging from 10 to 30 wt%. The study found that when a higher heating rate was used, AP decomposed violently. The ignition temperature of the optimized composite material was about 200 °C lower than that of pure nAl, and the weight gain rate was higher than that of uncoated nAl. Hao [19] prepared a core–shell-structured nAl/perfluorooctanoic acid (Fx) composite material (nAl@Fx). Differential scanning calorimetry (DSC) results showed that the instantaneous heat flow of nAl@Fx composite material at 633 °C was 155 W/g, significantly higher than the 15.4 W/g at 573 °C for nAl. Combustion tests indicated that the maximum combustion flame temperature of nAl@Fx composite material reached 1366 °C, also higher than the 1198 °C of raw-material nAl. Wang [20] prepared nAl/polymer of perfluorosulfonic acid (PFSA) nanocomposite energetic materials by using an average particle size of 50 nm for nAl and a content of 30 wt% aluminum powder, and compared it with an Al/PTFE system of the same mass ratio. DSC results showed that the reaction exothermicity of the nAl/PFSA system was 2675 J/g, approximately three times that of the nAl/PTFE system at 961.8 J/g, with a starting reaction temperature of 370 °C, much lower than the 463.8 °C of the nAl/PTFE system. PFSA is more effective in combustion of aluminum powder than PTFE. Shi [21] used layer-by-layer assembly technology to alternately coat copper-trimesic acid(Cu(BTC)) and iron-trimesic acid(Fe(BTC)) on the surface of nAl, preparing a core–shell-structured n-Al@Cu(BTC)/Fe(BTC) nano-aluminum thermal agent. The preparation process is shown in Figure 2. Research results indicate that compared to pure nAl, the composite material exhibits more intense combustion reactions, faster burning rates, and a significantly lower oxidation reaction temperature.

Nitrocellulose (NC) has high energy density. Wang [22] prepared Al/NC composites using electrostatic spray technology, employing aluminum powder with a particle size of less than 50 nm, containing about 70% active aluminum by mass, and NC accounting for 10% by mass. Ignition test studies have shown that Al/NC composite particles prepared using the electrostatic spray method burn much more vigorously than untreated nano-Al particles, producing dazzling fireballs, with ignition delay time sharply reduced from 14 ms to 0.3 ms. Luo [15] investigated the effects of surface coating with polyethylene glycol (PEG) and fluorocarbon surfactant TF3721 (F–C surfactant) on the reaction characteristics of aluminum powder. The aluminum powder used was at the nanoscale, with an active aluminum content of 89.70%. Combustion tests demonstrated that PEG/nAl composite particles and F–C/nAl composite particles both exhibit better combustion performance compared to pure nAl. Pure nAl cannot be ignited directly, while the ignition delay time for PEG/nAl composite particles is 0.95s, and for F–C/nAl composite particles, it is 1.20s. The burning rate for PEG/nAl composite particles is 0.050 cm·s^−1^, and for F–C/nAl composite particles, it is 0.065 cm·s^−1^, indicating that F–C/nAl composite particles have a higher burning rate and exhibit more intense combustion phenomena. Zhu [23] studied the effect of KBH4 surface coating on the combustion assistance of aluminum powder. The average particle size of the aluminum powder used was 50 nm, with a purity of 80.8%. The study found that when the KBH_4_ content was 3%, the initial ignition temperature was 429 °C, which was 8.54% lower than that of pure aluminum powder. The maximum combustion temperature was 1404 °C, which was 1.52% higher than that of pure aluminum powder. Zeng [24] modified aluminum nanoparticles by grafting them onto high-energy glycidyl azide polymer (GAP) in situ to prepare composite materials. The diameter of the aluminum powder used was approximately 50 nm, with an activity of 93.6%. On this basis, (Al@GAP)/fluorine composite materials were also prepared. Ignition tests showed that GAP could shorten the ignition time of aluminum powder, and (Al@GAP)/fluorine composite materials had even shorter ignition times and more intense flames. Mulamba [25] used polytetrafluoroethylene (PTFE) to coat nAl with an average particle size of 80 nm. Combustion tests showed that the combustion rate of nAl coated with PTFE significantly increased due to the exothermic reaction between A1_2_O_3_ on the aluminum powder surface and PTFE. Cheng [26] prepared nickel-coated nano-A1 composite particles using a chemical plating method in a medium containing nAl through the redox reaction of nickel acetate (Ni(Ac)_2_·4H_2_O) with sodium hydroxide (NaOH). Thermogravimetric analysis (TG) revealed that the oxidation onset temperature of the prepared composite particles was 190–260 °C lower than that of pure nAl. Qiu [27] also prepared nickel-coated nAl composite particles using a similar method, with similar results. Kaplowitz [28] coated Fe_3_O_4_ on the surface of nAl through gas-phase reactions and formed an aluminum-thermal agent with CuO. T-Jump ignition tests showed that the ignition temperature of the aluminum-thermal agent formed by surface-coated aluminum powder was 973 K, while that of the uncoated aluminum powder was 1076 K, indicating higher reactivity of nAl after Fe_3_O_4_ coating. Yang [29] prepared nAl@polyvinylidene fluoride (PVDF) microspheres using the electrospray deposition method, with an average aluminum powder particle size of 50 nm and PVDF contents of 5%, 10%, and 15 wt%. Combustion tests and thermogravimetry–differential scanning calorimetry analysis showed that compared to raw-material nAl, nAl@PVDF had better combustion performance and a more intense exothermic process (sharper exothermic peaks). Li [30] synthesized fluorinated polydopamine (PF) and coated it on nAl to prepare a core–shell-structured composite material. The optimized formulation had a combustion rate (196.4 mm s^−1^) 8.1 times higher than that of the original nAl (24.2 mm s^−1^) and 3.6 times higher than that of the physically mixed sample (54.7 mm s^−1^). The energy-containing composites of micro-sized Al and nano-sized Al powder modified by the hexafluoropropylene and vinylidene fluoride dipolymers (VitonA) were fabricated by an evaporation solvent method by Chen [31]. Compared with Al, the Al@VitonA composites display superior ignition and combustion (IAC) performance, characterized by the decrease in ignition delay time, the improvement of combustion intensity, the expansion of fame area, and the decrease in agglomeration behavior.

Energetic metal–organic frameworks (EMOFs) constitute an emerging research hot-spot material. He [32] used EMOFs as precursors for metal oxides and as oxidants in conventional nano-aluminum heat sources to prepare metastable nano-composites nAl@EMOFs. The average particle size of the aluminum powder was 80 nm. EMOFs are the reaction products of 5,5-bis(azinol-1,1-diazole)tetrahydroxyl (DHBT) and Cu(NO_3_)_2_·3H_2_O. The results showed that the ignition temperature of nAl@EMOFs was 301.5 °C, much lower than the 578 °C of nAl, indicating that this structure provides excellent combustion assistance for aluminum powder.

The above studies show that the surface coating of nAl powder can significantly improve the chemical reaction performance of nAl, greatly improve the application feasibility of nAl in explosives, and promote the application process of nAl in explosives and propellants.

### 2.3. Increasing the Reaction Degree of nAl

The degree of reaction of aluminum powder is one of the keys to its effectiveness in explosives. It is evident that the higher the reaction degree, the more energy advantages aluminum powder can exert. The most commonly used method to characterize the reaction degree is total heat of reaction. Yan [33] used fluororubber F2602 with a fluorine content of 66% and coated nAl on its surface, with fluororubber mass contents of 5%, 10%, and 15%. Differential scanning calorimetry (DSC) studies found that the heat release of uncoated samples was 6451 J/g, while the heat release of the three coated samples was 6902, 7892, and 9259 J/g, respectively, representing increases of 6.99%, 22.34%, and 43.53% compared to the uncoated samples. As the fluororubber content increased, the total heat release also significantly increased. Lu [34,35,36,37,38,39,40,41,42,43] coated nAl using polytetrafluoroethylene (PTFE), fluororubber F2311, fluororubber F2314, polyvinylidene fluoride (PVDF), tetrafluoroethylene-hexafluoropropylene-vinylidene fluoride copolymer (THV), fluorinated acrylic ester (PFDMA), perfluorododecane acid (C_11_F_23_COOH), perfluorotetradecane acid (PFTD), and fluorinated polyurethane (FPU), and similar results were obtained. He [44] prepared highly stable Al@TAM (M = Cu, Fe, Co, Bi) composites by coordination of tannic acid (TA) and metal ions on the surface of nAl. DSC results and product analyses show that the CuO/Al@TAM nanothermites exhibit higher energy release and reaction extent than that of CuO/Al; the total heat release of CuO/Al is 946 J/g, whereas the total heat release of CuO/Al@TACu and CuO/Al@TACo is 1202 J/g and 1254 J/g, respectively, representing an increase of about 30%.

**Figure 3:**
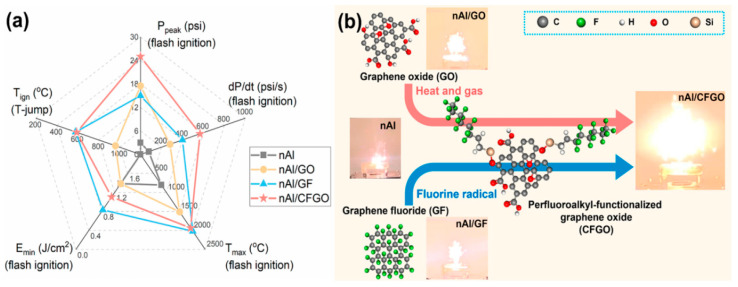
Energetic properties of nAl with different functionalized graphene additives [46]. (**a**) Comprehensive evaluation of the energetic performance of nAl with various functionalized graphene additives (T_ign_: ignition temperature in T-jump, E_min_: the minimum ignition energy of flash ignition, T_max_: the maximum combustion temperature achieved during the burning of flash-ignited samples in the air, P_peak_: the maximum pressure rises of the flash-ignited samples in a constant-volume vessel, dP/dt: the pressurization rate of the flash-ignited samples). (**b**) The molecular structure of different additives and their effects on nAl combustion and the burning phenomena of nAl with functionalized graphene additives.

Kim [45,46] coated nAl with polytetrafluoroethylene (PTFE), which serves both as a protective layer and an oxidizing agent. The study found that the weight-burning calorific value of the composite material increased from 0.88 kJ/g to 4.80 kJ/g, more than four times higher than that of pure aluminum. Jiang [46,47] modified graphene by functionalizing it with perfluorooctyltriethoxysilane (CFGO) using aluminum powder with a nominal particle size of 70 nm and an active aluminum content of 72.5 wt% to prepare nAl/CFGO (80/20 wt%) composite materials. nAl/GO and nAl/GF were also prepared at the same mass ratio for comparison. Constant volume ignition tests showed that nAl/CFGO exhibited the most intense combustion and the highest combustion temperature, with peak pressure and pressure rise rate increasing by more than seven times and six times, respectively, compared to the original aluminum powder. Figure 3 provides a comprehensive evaluation of the energy performance of nAl under different functionalized graphene additives, the molecular structures of the additives and their impact on nano-aluminum combustion, as well as the combustion phenomena of nano-aluminum with functionalized graphene additives. Additionally, the research team modified nAl with perfluoroalkylsilanes and obtained similar results.

Tang [48] explored the mechanism for significantly enhancing the reaction degree with suitable encapsulation materials. The study used fluorinated polyurethane (FPU) as a binder to prepare energetic composites containing aluminum nanoparticles (average particle size 100 nm). The thermal burst test showed that when the fluorine content in the composite was only 3.70 wt%, the thermal burst increased from 1685 kJ kg^−1^ in the non-fluorinated system to 3222 kJ kg^−1^, an increase of 91.2%. The authors’ calculations indicated that if the additional thermal burst was solely due to the formation of AlF_3_, it would be only 482 kJ kg^−1^. Therefore, the fluorine in the fluorinated material not only undergoes oxidation but also acts as a catalyst. When the fluorine content in the composite increased to 4.55%, 5.24%, 3.02%, and 6.79%, the thermal burst values were 2973, 2926, 3032, and 3037 kJ kg^−1^, respectively, with the thermal burst increasing as the fluorine content increased.

The above studies show that the suitable and appropriate amount of surface coating material has a very great influence on the reaction degree of nAl, which can even increase the heat release of raw aluminum powder several times. The use of fluorine-containing materials as surface coating modification materials is the key research direction of nAl surface coating modification.

## 3. Effect of Surface-Coating nAl on Energy Characteristics of Explosives and Propellants

### 3.1. Increasing the Energy Performance of Explosives

**Figure 4:**
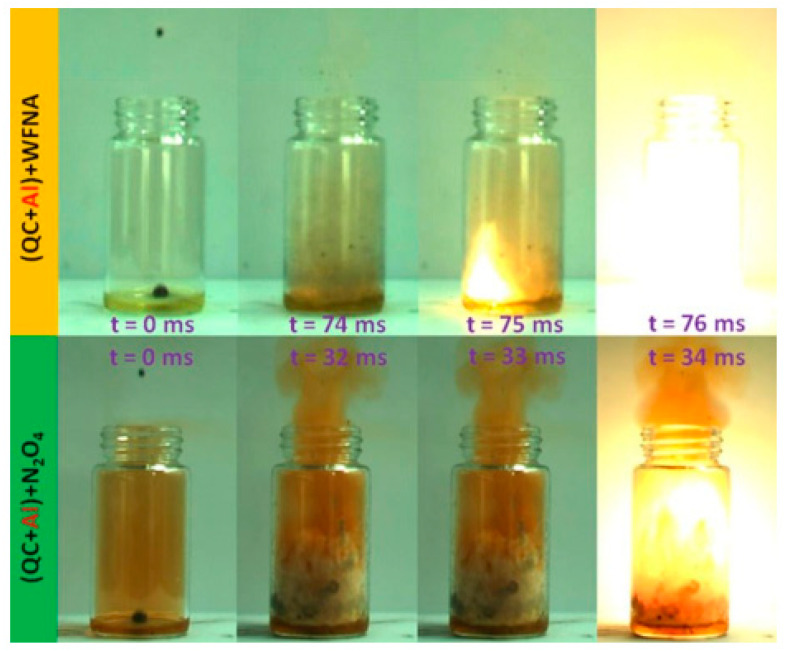
Ignition process of QC/WFNA and QC/N_2_O_4_ in the presence of Al NPs coated by oleic acid [47].

The impact of surface-coating nAl on explosive energy has also been explored by researchers. E. Xiu [49] used 0.25 w% oleic acid to coat nAl and added it to two self-made fuels, HD-01 and HD-03. The study found that when the nAl particle content was 30 wt%, the volumetric calorific values of the two suspension fuels were 44.1 MJ/L (Al/HD-01) and 45.4 MJ/L (Al/HD-03), respectively, which represented an increase of 12% compared to pure HD-01 fuel and 3% compared to pure HD-03 fuel. Additionally, ignition tests showed that the ignition delay for quadricyclane (QC)/white fuming nitric acid (WFNA) was 98 ms, while that for quadricyclane/N_2_O_4_ was 29 ms. When 0.25 wt% nAl particles were added, the ignition delays for both propellants could be reduced to 75 ms and 33 ms, respectively. Figure 4 shows the spontaneous ignition processes of QC/WFNA and QC/N_2_O_4_ after adding oleic-acid-coated aluminum nanoparticles, indicating that the modified nAl has a certain combustion-promoting effect on energetic material systems.

The activity of nAl is very high, and the reactivity of the redox reaction is better than that of HD-01 and HD-03 fuel and QC, while the stability of nAl coated with oleic acid is better than that of nAl. When nAl coated with oleic acid is added to HD-01 and HD-03 fuels and QC/WFNA and QC/N_2_O_4_ systems, the reactivity of the original system is increased, so the reaction is more complete, CO_2_ is generated, the reaction heat is higher than that of the original system, and the ignition delay time is also reduced. In a word, the application of surface-coated nAl to mixed explosives can improve the energy of explosives to a certain extent.

### 3.2. Increasing the Propellant Burning Rate and Regression Rate

The burning rate and regression rate of propellants significantly affect their application. Researchers have studied the regression rate of propellants by surface coating with nAl. The burning rate of propellant can be measured by the standard method described in [41,50]. Qin [51] prepared a composite material by surface-coating nAl on fluorine-containing materials (the specific components of the fluorine-containing materials and the aluminum powder particle size were not specified), which was applied to hydroxyl-terminated polybutadiene (HTPB) propellant. The mass content of aluminum powder on the surface was 10%, and it was compared with formulations without aluminum powder. Combustion test results showed that the migration rate of nAl-coated fuel on fluorine-containing materials increased by about 13% compared to the migration rate of fuel without the composite material. Sossi [50] obtained similar results, systematically studying the effects of nAl-coating on fluorinated elastomer surfaces and HTPB surfaces on the combustion performance of propellants. It was found that compared to pure HTPB, nano-Al particles coated on the surface significantly improved the ballistic performance of the formulation, with both coating systems showing an increase in instantaneous migration rates. Uhlenhake [41] prepared Al/THV composites with an average aluminum powder particle size of 80 nm and a purity of 70 wt%. They added the Al/THV composites to the AP/HTPB propellant system. When the composite material accounted for 5 wt%, the burning rate was 2.1 times that of the original propellant. When the composite material accounted for 15 wt%, the burning rate was 4.7 times that of the original propellant. In addition, the team also prepared Al/PVDF films, which showed similar effects [42].

The above research results show that the application of surface-coated nAl in propellant can obviously increase the burning rate and regression rate of propellant, mainly due to the high reactivity of nAl after surface coating.

### 3.3. Improving the Reactivity of Composite Energetic Materials

The reactivity of energetic composites significantly influences their application, determining both the feasibility and effectiveness of their use. Yao [52] studied the thermal decomposition characteristics of oleic acid (OA)-surface-coated nAl (nAl@OA) and uncoated aluminum powder. DSC results show that for the composite energetic material system composed of nAl and RDX before and after oleic acid surface coating, regardless of the heating rate at 5, 10, 15, 20, or 25 °C/min, the peak temperature of the thermal reaction in the oleic-acid-coated nAl composite material system is lower than that of the uncoated system, indicating that the reactivity of the composite material system containing oleic-acid-coated aluminum powder can be adjusted.

Obviously, the application of surface-coated nAl in energetic composites can adjust the reactivity of energetic composites and increase the application scenarios of surface-coated nAl.

## 4. Effect of Surface-Coating nAl on the Safety Performance of Explosives and Propellants

### 4.1. Optimizing Compatibilities

Compatibility directly determines whether a material can be used in explosives or propellants. Li [53] selected glycidyl azide ether (GAP) to surface-coat and modify nAl (nAl@GAP). Studies have shown that GAP not only prevents the oxidation and deactivation of nAl but also enhances its compatibility with propellant components. When the coated particles nAl@GAP are added to ammonium dinitramide (ADN), the decomposition temperature of the mixed material is significantly higher than when using uncoated nAl formulations. Therefore, the surface coating of nAl can improve the compatibility of the components of the explosives.

### 4.2. Reducing Mechanical Sensitivities

Mechanical sensitivities determine the safety performance of explosives during production, transportation, and storage. Li [54] prepared self-assembled materials with a Viton@FOX-7@Al core–shell structure using spray drying and compared them with FOX-7/Viton@Al using aluminum powder particles with a size of 50–100 nm. The study found that under the same conditions, the thermal activation energy of the Viton@FOX-7@Al material was lower than that of the FOX-7/Viton@Al material, and its mechanical sensitivities were also reduced. It can be seen that the mechanical sensitivities of energetic system can be reduced by preparing surface-coated nAl with appropriate technology.

## 5. Effect of Surface-Coating nAl on the Process Properties of Explosives and Propellants

An important application direction for surface coating is to optimize the process properties of aluminum-containing explosives. Metal–hydrocarbon slurries can be used in rocket engines, offering particular advantages in thrust, missile range, and operability. However, due to the much higher density of metal materials compared to liquid fuels, the issue of metal particle settling is the greatest challenge in their application [55].

Wu [56] prepared various large-molecular-chain-modified aluminum nanoparticles using the liquid reflux heating method and applied them to the suspension of tetrahydrodicyclopentadiene (named as JP-10)-based fluid fuel. The research results showed that Al@Span-65 material can achieve a stable dispersion time of over one month for the JP-10 and nAl mixed system. Wang [57] coated nAl with oleic acid, resulting in a uniformly distributed particle size of about 20 nm. For nAl with an oleic acid mass ratio of 1%, the mixture remained unchanged after six months of static storage when applied to JP-10. E. Xiu [58] obtained similar results. Chen [59,60] found that Al@Tween-85 material can effectively stabilize the settling of aluminum powder in the nAl-JP-10 system, and that oleic-acid-coated nAl has a similar effect on JP-10. Gan [61] prepared Al@Span-80 material, which can significantly stabilize the settling of aluminum powder in the nAl–n–heptane system. Kim [62] prepared nAl coated with oleic acid (OA), and added the modified nAl particles to kerosene, resulting in a uniformly suspended solution.

Obviously, the surface coating of nAl can optimize the process performance of metal–hydrocarbon slurry fuel and expand the service life of such materials.

## 6. Effect of Surface-Coating nAl on Other Properties of Explosives and Propellants

### 6.1. Reducing Hygroscopicity

Hygroscopicity affects the storage performance of explosives and propellants. If the hygroscopicity of a certain explosive formula is too high, its safety, energy, and mechanical properties will be seriously affected. Li [63] prepared nAl with an average particle size of about 100 nm by electrodetonation, and coated it with hydroxy-terminated glycidyl azide ether (GAP) with a surface coating thickness of about 3.7–5.5 nm. The relative humidity of the test environment was 90%, and the test temperature was 30 °C. The test results show that between 20 h and 140 h, the hygroscopity of non-surface-covered nAl increased with the increase of time, from the initial 1.3% to 2.1%, while the hygroscopity of GAP-surface-covered nAl was initially 0.33%, and at its highest reached only 0.37%. GAP-protected nAl has a significant effect on the hygroscopic properties of explosives. It can be seen that the surface coating of nAl can improve the hygroscopicity of energetic systems and increase the application scenarios of aluminum powder.

### 6.2. Optimizing Combustion Products of Propellants

**Figure 5:**
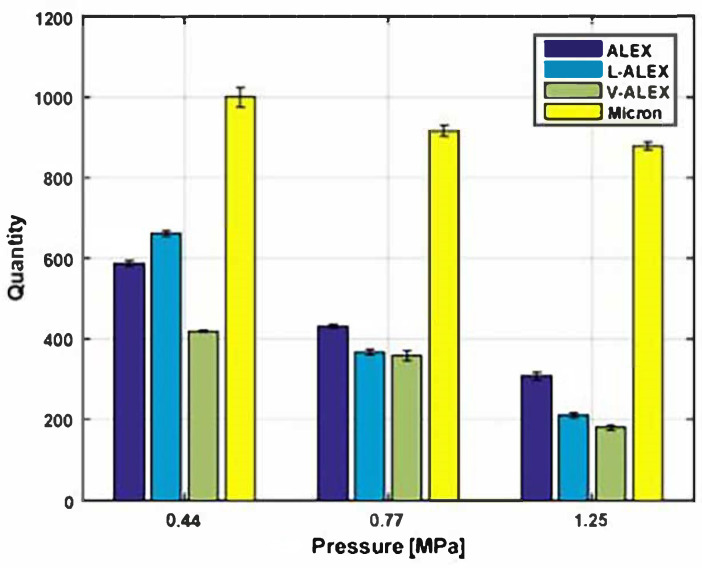
Agglomerate quantity vs. operating pressure (the powders are ALEX, uncoated nano-aluminum; V-ALEX, a fluoropolymer (Viton)-coated nano-aluminum; and L-ALEX, a stearic-acid-coated nano-aluminum) [64].

The combustion products of propellants have a significant impact on their propulsion efficiency. If the combustion products of the propellant tend to agglomerate or form large aggregates, it indicates incomplete combustion and poor propulsion efficiency. Cohen [64] used fluororubber Viton (Viton) and stearic acid surface-coating of nano-aluminum (nAl) in propellants, with the formulation composition being 20% HTPB/65% AP/10% micron aluminum/5% nAl. Combustion tests showed that compared to propellants containing only micron powders, those with surface-coated nano-powder exhibited up to a 60% reduction in aggregate volume, and were superior to formulations containing uncoated aluminum powder. Figure 5 illustrates the agglomeration levels of different sizes of aluminum powder under various pressures. Therefore, replacing part of the micron aluminum powder with surface-coated nano-aluminum in solid propellants can significantly reduce the loss in propulsion efficiency due to agglomeration.

Therefore, coating nAl with suitable additives can significantly reduce the combustion products of propellants and reduce the agglomeration of combustion products, thus greatly improving the energy efficiency of propellants.

The characteristics of surface-coated nAl and its influence on the properties of explosives are summarized in Table 1.

## 7. Conclusions

(1) Surface-coating nAl can protect its activity, improve its reactivity, and increase its reaction heat, among many other advantages.
(2) The application of surface-coated nAl in explosives or propellants can increase the energy performance of explosives and the burning rate of propellants.
(3) From the perspective of safety, the application of surface-coated nAl in explosives can improve the compatibility between nAl and energetic materials and reduce the mechanical sensitivity of energetic mixtures.
(4) From the perspective of process, the application of surface-coated nAl in the metal–liquid fuel system can improve the stability of nAl–hydrocarbon slurry fuel and enhance the service life of such materials.
(5) The application of surface-coated nAl in explosives or propellants can also reduce the hygroscopicity of energetic composites and greatly reduce the agglomeration of propellant combustion products, thus reducing the loss of propulsion efficiency caused by agglomeration.

In practical application, the vast majority of micron-sized aluminum powder has an active aluminum content of more than 98%. Although a large number of researchers have analyzed the influence of surface-coated modification of nAl from multiple perspectives, the existing literature still has not found a process and material that can make the active aluminum content of surface-coated nAl exceed 98%. The authors suggest that researchers should strengthen research from this perspective. If the content of nAl active aluminum modified by surface coating can exceed 98% through appropriate technology and materials, close to or even consistent with micron-sized aluminum powder, it will bring a disruptive technological revolution to explosives and propellants formulations.

## Figures and Tables

**Table 1:** Characteristics of surface-coated nAl and its influence on the properties of explosives.

	Specific Impacts	Coating Material Used	Characteristics	Ref.
Effect of surface coating on nAl	Protecting the activity of nAl	PS, HTPB-TDI, ODA, paraffin wax, perfluorotetradecanoic acid, epoxy resin, TMPTA, palmitic acid	These protect the activity of nAl and reduce the oxidation rate of nAl.	[8,9,10,11,12,13]
		Boron powder	Besides protecting the activity of nAl, it can also increase the reaction heat of the system.	[14]
		PEG	The nAl reaction temperature is shifted backward.	[15]
	Improving the reactivity of nAl	Carbon, nickel, EMOF, AP, PFSA	The exothermic reaction is advanced or the ignition temperature is reduced.	[16,18,20,26,27]
		KBH_4_, Fe_3_O_4_	Ignition delay time is shortened and reaction is more intense.	[17,24]
		Perfluorooctanoic acid	Increasing instantaneous heat flow	[19]
		Cu(BTC), PTFE, PF	Increasing the burning rate	[21,25,30]
		NC, PVDF, VitonA	Burning more violently	[22,29,31]
		Perfluorootetradecanoic acid, GAP	Increasing the maximum combustion temperature	[23,28]
	Increasing the degree of reaction of nAl	Fluorinerubber F2602, PTFE, fluorinerubber F2311, fluorinerubber F2314, PVDF, THV, PFDMA, C_11_F_23_COOH, PFTD, FPU, tannic acid	Increasing the total heat of reaction	[33,34,35,36,37,38,39,40,41,42,43,44,45,46,47,48]
Effect of surface-coating nAl on energy performance of explosives and propellants	Increasing the energy of mixed explosives	Oleic acid	Increasing the volume calorific value of suspended fuel	[49]
		Fluoroelastomers, fluorine-containing materials	Increasing the burning rate of propellants	[50,51]
	Increasing the burning rate or regression rate of propellants	THV, PVDF	Increasing the regression rate of propellants	[41,42]
	Adjusting the reactivity of composite materials	Oleic acid	Decreasing thermal reaction peak temperature	[52]
Effect of surface-coating nAl on the safety performance of explosives	Improving compatibility	GAP	Improving the compatibility of components in the formula of explosives and propellants	[53]
	Reducing mechanical sensitivities	FOX-7+Viton	Reducing the mechanical sensitivity of explosive system	[52]
Effect of surface-coating nAl on the process properties of explosives	Process properties	Span-65, Tween-85, oleic acid, Span-80	Optimizing the process properties of metal–hydrocarbon slurry fuel and expanding the service life of such materials	[54,55,56,57,58,59,60]
Effect of surface-coating nAl on other properties of explosives or propellants	Hygroscopicity	GAP	Improving the hygroscopicity of energetic system	[61]
	Combustion products of propellant	Viton+stearic acid	Aggregation is greatly reduced, and the loss of propulsion efficiency caused by agglomeration is significantly reduced.	[62]

## Data Availability

Data are contained within the article.
